# Metaviromic insights into the viral community associated with *Dendrobium catenatum*

**DOI:** 10.1007/s42770-025-01816-5

**Published:** 2026-01-28

**Authors:** Rogério Mercês Ferreira Santos, Lucas Yago Melo Ferreira, João Pedro Nunes Santos, Lucas Barbosa de Amorim Conceição, Giovanna Venas Oliveira, Cassio van den Berg, Eric Roberto Guimarães Rocha Aguiar

**Affiliations:** 1 Data Processing and Bioinformatics Laboratory (BIOINFO), Department of Biological Sciences (DCBIO), Universidade Estadual de Feira de Santana (UEFS), Avenida Transnordestina, No Number, Novo Horizonte, Feira de Santana, Bahia 44036-900 Brazil; 2 Center of Biotechnology and Genetics, Department of Biological Sciences, Universidade Estadual de Santa Cruz (UESC), Rodovia Jorge Amado, km 16, Ilhéus, Bahia 45662-900 Brazil; 3 Postgraduate program in Computational Modeling in Science and Technology, Department of Engineering and Computing, Universidade Estadual de Santa Cruz (UESC), Ilhéus, BA Brazil

**Keywords:** Mycoviruses, Orchidacea, Transcriptomics, EVEs, Bioinformatics

## Abstract

Metatranscriptomic analysis of public RNA-seq data from *Dendrobium catenatum*, an economically and culturally important orchid, revealed a highly diverse virome. Sixteen exogenous viral species were detected, representing seven families of RNA viruses, including positive-sense ssRNA viruses (*Botourmiaviridae*, *Mitoviridae*, *Narnaviridae*, *Endornaviridae*, and *Fusariviridae*) and double-stranded RNA viruses (*Phlegviridae* and *Partitiviridae*). In addition, seven endogenous viral elements (EVEs) were identified, with a predominance of *Mitoviridae*, suggesting historical viral integration events. The detected virome reflected complex interactions between plant-associated fungi and the host orchid, and many of detected viral families are commonly associated with fungal hosts, suggesting that several viruses may be of mycoviral origin, though their exact host remains to be confirmed. Phylogenetic analyses and compositional correlations point toward potential horizontal virus transfer events across kingdom boundaries, further complicating the ecological network of virus-host relationships. To explore virus-host adaptation, we also analyzed codon usage and dinucleotide/trinucleotide composition profiles, revealing clustering patterns that suggest co-evolutionary pressures and potential host-specific optimization of viral genomes. These molecular signatures enhance our understanding of how viruses may adapt to diverse hosts and underscore the complex evolutionary dynamics shaping the *D. catenatum* holobiont. These findings not only broaden our knowledge of orchid-associated viral diversity but also provide critical insights into virus evolution and cross-kingdom virome interactions.

**Supplementary Information:**

The online version contains supplementary material available at 10.1007/s42770-025-01816-5.

## Introduction

The study of orchid microbiota, particularly within *Dendrobium catenatum* species, is gaining significant attention due to its implications for plant health, ecology, and conservation [62]. Orchids, known for their intricate relationships with mycorrhizal fungi, represent a unique ecological niche where microbial interactions play a crucial role in nutrient acquisition and overall plant vitality [18]. The *Dendrobium* genus, composed of more than 1000 species, which includes *D. catenatum*, is particularly noteworthy because it is widely cultivated and has substantial economic importance in horticulture and traditional medicine [53]. Understanding the microbiota associated with these orchids can provide insights into their growth, resilience, and adaptability under various environmental conditions [20, 35].

Despite the recognized importance of the orchid microbiota, there remains a significant gap in our understanding of the specific microbial communities, particularly the virome associated with *D. catenatum* [48]. While studies have explored the fungal and bacterial components of orchid microbiomes, viral constituents have been largely overlooked. This lack of knowledge is concerning, because viruses can significantly influence host health and interactions within the microbial community. For instance, the presence of mycoviruses in fungal symbionts can affect the virulence of plant pathogens, potentially altering the dynamics of plant-fungal interactions [34]. Therefore, a comprehensive investigation into the virome of *D. catenatum* is essential to elucidate its role in the health and sustainability of these orchids.

Viruses can significantly influence host health and microbial community interactions, thereby affecting plant-fungal dynamics [45, 46]. Investigating the virome of *D. catenatum* is essential for comprehending its role in orchid health and sustainability, as viruses may play a dual role—either as pathogens that harm the plant or as symbionts that can potentially enhance plant fitness [52]. Recent advancements in high-throughput sequencing technologies present a promising opportunity to explore the viral landscape associated with orchids in a comprehensive manner. Such technologies enable researchers to uncover the complex interactions between viruses, fungi, and orchids, providing a deeper ecological understanding of these relationships [34]. By delving into the virome of *D. catenatum*, scientists can gain insights into how these viral entities affect the overall health and sustainability of orchid populations, which is particularly vital in the context of conservation efforts aimed at protecting these unique and often endangered species.

Moreover, the intricate relationships between orchids and their microbial partners extend beyond mere survival; they influence the overall genetic diversity and evolutionary trajectories of these plants [38]. For instance, recent studies have suggested that the endophytic microbiota can play a pivotal role in shaping the phenotypic traits of orchids, potentially enhancing their adaptability to environmental changes [2]. This phenomenon is particularly relevant for *D. catenatum*, as its ability to thrive across varied habitats may be closely linked to the diverse microbial communities it harbors within its tissues. Furthermore, understanding how specific microorganisms contribute to the resilience of orchids against pathogens could lead to innovative strategies for conservation and cultivation practices, ultimately supporting sustainable horticulture and preserving biodiversity [16].

The purpose of this article was to investigate the diversity of the virome associated with *D. catenatum* using bioinformatics tools to analyze public transcriptomic data. Our study explored the complex interactions among plant, fungal, and viral communities within *D. catenatum*, shedding light on patterns of virus–host coevolution and horizontal virus transfer across kingdoms. Ultimately, this work advances our understanding of orchid-associated viral diversity and provides a foundation for future research into the ecological and evolutionary implications of these intricate viral networks.

## Materials and methods

### Acquisition of RNA libraries

Publicly available RNA-seq datasets for *D. catenatum* were retrieved from the NCBI Sequence Read Archive (SRA). A total of 33 libraries, encompassing various tissue types (e.g., mature leaf, stem and young leaves) and conditions, including infection or co-infection with *Sclerotium delphinii*, were selected [21, 56, 61, 67]. Among these, 23 libraries were used as input for virus discovery while 10 were used as biological replicates to access independent RNA levels. Detailed library descriptions are provided in Supplementary File 1.

### Metatranscriptome assembly

The analysis of publicly available RNA-seq libraries followed a protocol adapted from [11] [11]. To identify viral sequences, RNAseq raw reads underwent quality assessment using FastQC (v0.11.9) [59] and trimming with Trimmomatic (v0.39) [4] to remove adapters and low-quality bases (Phred <20). Trimmed reads were mapped against *D. catenatum* genome (Acession: GCF_001605985.2), using Bowtie2 (v2.5.0) [23] to filter host-related sequences. Unmapped reads were de novo assembled using rnaSPAdes (v3.15.5) [42] with default parameters. Putative viral contigs were identified through sequence similarity searches using Diamond (v2.0.15) in BLASTx mode, with the NCBI Viral RefSeq database (release 218) as reference.

### Microbiome composition assessment

The assembled contigs were analyzed for taxonomic classification by comparing them with the reference database PlusPFP (version: 2022-06-07) on Kraken2 (Galaxy Version 2.1.3 + galaxy1) [60]. The resulting Kraken-report files (Galaxy Version 1.3.1) were then processed with Bracken v3.1 [26] to re-estimate abundance profile at both phyla and genus taxonomy levels.

### Identification of endogenous viral elements

We utilized the Galaxy online platform (https://usegalaxy.org/) to differentiate between potential Endogenous Viral Elements (EVEs) and exogenous viral species circulating in *D. catenatum*. We screened the reference genome (Acession: GCF_001605985.2), for viral sequences. Open Reading Frames (ORFs) were initially predicted using Getorf (v5.0.0.1) [43] with a length range of 100 to 6000 nucleotides. These ORFs were then aligned against the RefSeq viral protein database (release 218) using DIAMOND (v2.0.15) [6] in BLASTx mode. The results were carefully curated to retain high-quality matches and exclude sequences unrelated to viruses. To reduce redundancy, CD-HIT [25] clustering was applied at a 90% similarity threshold. The non-redundant sequences were further analyzed using NCBI’s BLASTn and BLASTx to identify similarities with known viral and non-viral proteins.

Therefore, for each candidate ORF we manually inspected the top 30 BLASTn and BLASTx hits, retaining sequences that met the following criteria: i) Presence of at least one viral hit within the top 30 BLASTx matches; and ii) detection of a conserved viral domain. If no conserved viral domain was detected, sequences were retained only when viral hits occurred within the top 10 BLASTx results. Candidates failing both criteria were excluded from downstream analyses.

### Characterization of putative exogenous viral sequences

Only contigs longer than 500 nucleotides were selected for further analysis. ORFs were predicted using the ORFfinder [44] tool and functional annotation was conducted with InterPro [3], HMMER [13, 41], and CD-Search [29] tools to identify conserved protein domains. Putative viral contigs were further characterized using BLAST searches against NT and NR databases (release 04/2024). A summary of the similarity search results obtained at the nucleotide and amino acid levels for each viral sequence can be found in Supplementary File 2.

### Phylogenetic analysis

Sequences encoding polymerases or polyproteins were used for phylogenetic tree construction. Additional protein sequences from families such as *Narnaviridae*, *Botourmiaviridae*, *Mitoviridae*, *Fusariviridae*, *Fusagraviridae*, *Orthototiviridae*, *Megabirnaviridae*, *Phlegiviridae*, *Trichomonasviridae*, *Victoriviridae*, and *Endornaviridae*, as well as other families within the Lenarviricota and Duplornaviricota phyla, were included based on similarity and references from the International Committee on Taxonomy of Viruses (ICTV). Global alignments were performed using MAFFT [22] with default parameters. ModelTest [40] was used to determine the best substitution model, and maximum likelihood phylogenetic trees were generated with 1000 bootstrap replicates using the CIPRES Science Gateway [31].

### RNA abundance analysis

The transcriptional activity of virus-derived sequences, along with *D. catenatum* genes retrieved from the current genome annotation (Acession: GCF_001605985.2), was analyzed using Salmon (v1.10.3) [36]. The genes, actin 7 (ACT7), Histone H1(H1), and cytochrome c oxidase subunit 2 (COX2) were employed as endogenous controls. Detailed results of viral transcriptional activity are provided in Supplementary File 3.

### Codon usage and nucleotide composition

Codon usage bias, which varies among species, imposes selective pressure on viruses, as the lack of matching tRNAs can hinder the efficient translation of viral proteins. Leveraging this concept, we inferred potential viral hosts by analyzing codon usage patterns and dinucleotide composition. ORFs were identified using ORFfinder.

To refine our dataset, a selection step was applied to the assembled contigs: sequences were realigned using DIAMOND against the NCBI fungal reference database. Contigs showing over 90% identity with fungal sequences were retained, and only those corresponding to species with available mitochondrial genome data were included—based on the observation that several viruses were associated with mitochondria. Notable exceptions were *Agraothelium delphinii* and *A. rolfsii*. Although the former was not among the species with deposited mitochondrial genomes, its presence in some libraries suggested it could represent a relevant host and a known orchid pathogen. The complete list of selected species is presented in Supplementary File 4.

Subsequent analyses of codon usage were performed using CUSP, while dinucleotide frequencies were calculated with COMPSEQ—both part of the EMBOSS suite [43]. Correlation analyses were then conducted to compare the genomic profiles of hosts and viruses, enabling the visualization of codon and dinucleotide usage trends across both groups.

## Results

### Assessment of microbial composition

**Fig. 1:**
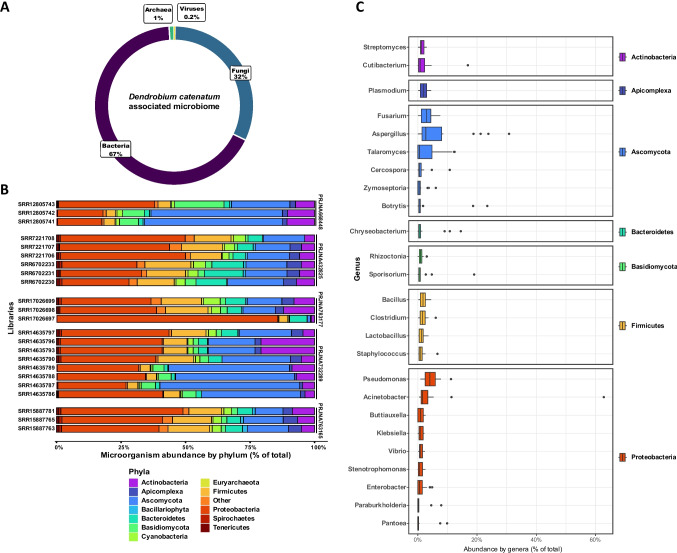
Microbial community profiling in *D. catenatum* RNA-seq datasets. **A** Taxonomic composition of the total microbiome across all datasets, showing the relative abundance of Bacteria, Fungi and Archaea. **B** Bar plots representing the relative abundance of microbial taxa (at the phylum level) in individual RNA-seq libraries, highlighting the diversity of fungal and bacterial communities, with minor representation of archaea. **C** Boxplots showing the overall relative abundance of microbial taxa at the genus level for the 25 most enriched genera across all analyzed libraries

The microbiome analysis of *Dendrobium catenatum* revealed a taxonomically rich and complex microbial assemblage encompassing bacteria, fungi, archaea, and viruses (Fig. 1A). It is important to note that the majority of the analyzed RNA-seq datasets were not generated with microbiome profiling in mind, and only the samples from project PRJNA732289 included surface sterilization of plant tissues prior to library preparation. Among the detected taxa, bacteria dominated the overall community composition, representing approximately 67% of the detected taxa, followed by fungi at 32%, with archaea and viruses contributing minor proportions (1% and 0.2%, respectively). This pattern was consistent across most libraries, indicating a stable bacterial-fungal core microbiome.

At the phylum level, the bacterial community was largely composed of Proteobacteria, Actinobacteria, Bacteroidetes, and Firmicutes, which collectively accounted for the majority of bacterial reads (Fig. 1B). Actinobacteria was the most prevalent group, with the library SRR17026697 showing nearly 80% abundance, primarily represented by the *Acinetobacter* genus (Fig. 1B and C). Firmicutes were also detected at variable levels, particularly in samples from project PRJNA783177, where they were more abundant in control and methyl jasmonate–treated samples but markedly reduced in those inoculated with *Sclerotium delphinii*.

The fungal community was primarily dominated by Ascomycota and Basidiomycota, although some samples—particularly within PRJNA732289, inoculated with *S. delphinii*—showed increased representation of these phyla, with notable enrichment of the genera *Aspergillus*, *Zymoseptoria*, *Botrytis*, and *Sporisorium* (Fig. 1B and C). Archaeal sequences were relatively scarce and mainly affiliated with Euryarchaeota and Crenarchaeota. More detailed *D. catenatum* microbiome composition can be seen in Supplementary File 5.

### Dendrobium catenatum-associated virome

#### Endogenized viral sequences

One of the major challenges in virus discovery is distinguishing between endogenous viral elements (EVEs) and exogenous viral sequences [5]. To address this issue, we conducted a thorough screening of the *D. catenatum* genome. A total of 7 EVEs, including RdRp and nucleoprotein sequences, were identified as integrated elements into *D. catenatum* genome. These EVEs were distributed across different viral families *Mitoviridae (5)*, *Amalgaviridae (1)* and *Rhabdoviridae (1)*, with lengths ranging from 120 to 702 nt. These sequences were subsequently used as negative controls for identifying putative exogenous viral sequences. A detailed overview of the identified EVEs is provided in Supplementary File 6.

#### Exogenous viral community associated with *D. catenatum*

**Fig. 2:**
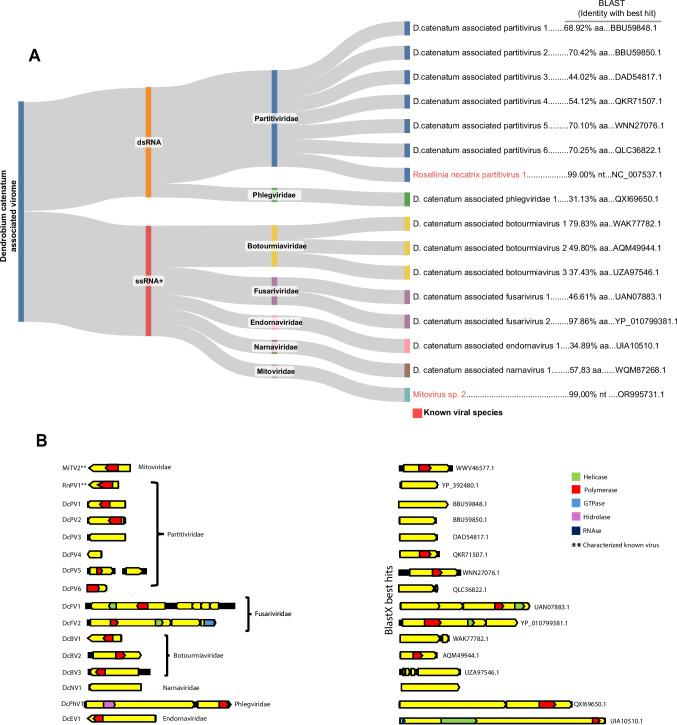
Diversity and genomic characterization of *D. catenatum*-associated viruses. **A** Sankeyplot visualization of the viruses associated with *Dendrobium catenatum*, classified into dsRNA and ssRNA families. On the left, the host species (*D. catenatum*) is displayed; in the center, the identified viruses are grouped by their respective families (e.g., *Partitiviridae*, *Botourmiaviridae*, and *Narnaviridae*). To the right, the best BLAST similarity results with corresponding identity values are shown. **B** Genomic characteristics and conserved domains of the identified viral sequences (left) alongside their best BLASTx hits (right). Sequence lengths are represented by the extent of the lines, with open reading frames (ORFs) shown as yellow boxes. Conserved domains are highlighted in distinct colors based on their putative functions. The identified viruses include Mitovirus sp. 2 (MiTV2), Rosellinia necatrix partitivirus 1 (RnPV1), Dendrobium catenatum-associated partitivirus 1–6 (DcPV1–6), Dendrobium catenatum-associated botourmiavirus 1–3 (DcBV1–3), Dendrobium catenatum-associated fusarivirus 1 and 2 (DcFV1–2), Dendrobium catenatum-associated narnavirus 1 (DcNV1), Dendrobium catenatum-associated phlegivirus 1 (DcPhV1), and Dendrobium catenatum-associated endornavirus 1 (DcEV1). Previously characterized viral sequences identified in *D. catenatum* are denoted with (**)

A detailed analysis of the contigs’ best hits revealed a diverse array of viruses, encompassing both positive-sense single-stranded RNA (ssRNA+) and double-stranded RNA (dsRNA) viruses. At the family level, the ssRNA+ viruses included representatives from *Botourmiaviridae*, *Mitoviridae*, *Narnaviridae*, *Endornaviridae*, and *Fusariviridae* (Fig. 1A). The dsRNA viruses were associated with elements related to the *Phlegviridae* and *Partitiviridae* families (Fig. 2A).

##### Characterization of known viral species

Two contigs showed sequence similarity to known viral species at both the nucleotide and amino acid levels, with coverage exceeding 98% and identity greater than 90%. The first, spanning 1825 nt, was closely related to Mitovirus sp. 2 (MiTV2), an unclassified member of the *Mitoviridae* family. It contained an incomplete open reading frame (ORF) of 2067 nt, which featured the Mitovir_RNA_pol (accession: cl05469) superfamily domain, a characteristic domain typically found in members of this family (Fig. 2B). The second contig, 2611 nt long, was related to Rosellinia necatrix partitivirus 1 (RnPV1) and presented a complete ORF of 1578 nt, which contained a ps-ssRNAv_RdRp-like superfamily domain (accession: cl40470) (Fig. 2B).

##### Description of novel viral species

Fifteen contigs exhibited sequence similarity to known viral species exclusively at the amino acid level. These contigs were associated with at least six viral families spanning four distinct viral phyla: Pisuviricota (*Partitiviridae* and *Fusariviridae*), Lenarviricota (*Botourmiaviridae* and *Narnaviridae*), Duplornaviricota (*Phlegviridae*), and Kitrinoviricota (*Endornaviridae*).

##### Pisuviricota

Viruses in the *Partitiviridae* family possess bi-segmented double-stranded RNA (dsRNA) genomes ranging from 3 to 4.8 kb. This family is divided into five distinct genera, with a diverse host range that includes plants, fungi, and protozoa. Replication occurs in the cytoplasm, with the two genome segments serving specialized functions: dsRNA1 encodes the RNA-dependent RNA polymerase (RdRp), while dsRNA2 typically encodes the coat protein [54].

**Fig. 3:**
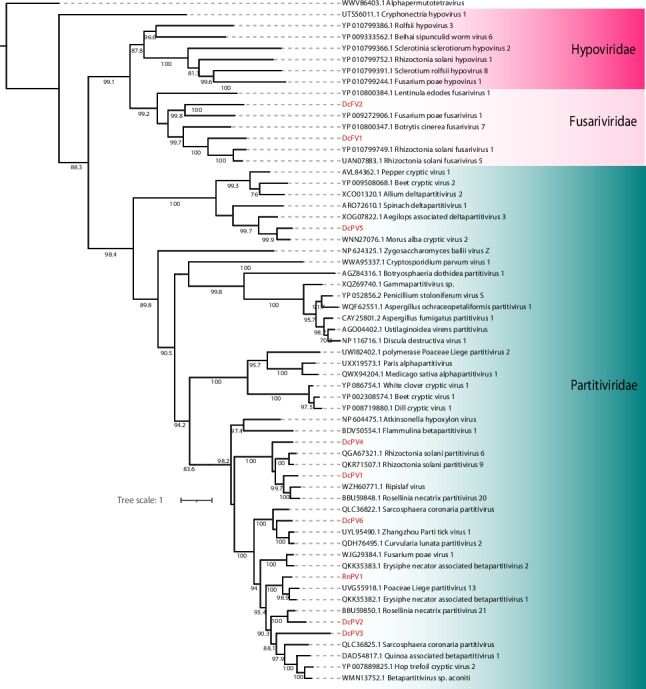
Phylogenetic reconstruction of assembled sequences related to the Psiviricota phylum: Phylogenetic analysis, conducted using ModelTest-NG based on the Akaike information criterion (AIC), determined Blosum62 + F as the optimal evolutionary model. Bootstrap values were generated from 1000 replicates. Values below 70% are not displayed

A total of seven contigs associated with the *Partitiviridae* family were identified, including four complete polymerase-encoding sequences, two incomplete polymerase sequences, and one coat protein sequence. These contigs ranged in length from 851 to 2409 nt and exhibited sequence similarity exclusively at the amino acid level. Most of the contigs displayed conserved domains consistent with their closest matches, such as the ps-ssRNAv_RdRp-like domain (accession: cl40470). Exceptions included one incomplete polymerase contig and the coat protein contig, which lacked identifiable conserved domains (Fig. 2B). Phylogenetic analysis of the polymerase-encoding ORFs confirmed that these sequences belonged to the *Partitiviridae* family, with only one sequence classified at the genus level as Deltapartitivirus. The identified sequences were designated Dendrobium catenatum-associated partitivirus 1 to 6 (DcPV1–6) (Fig. 3).

The *Fusariviridae* family comprises mono-segmented, positive-sense RNA viruses with genome lengths ranging from 5.9 to 10.7 kb. Their bicistronic genomes typically encode two proteins: the RNA-dependent RNA polymerase (RdRp) and an RNA helicase within the larger ORF, while the product of the second ORF remains poorly characterized. These viruses are predominantly associated with fungal hosts, although some studies have suggested oomycetes as alternative hosts [8, 28].

In this study, two assembled transcripts demonstrated amino acid-level similarity to members of this viral family. The first transcript, 9257 nt in length, contained a single large ORF harboring the ps-ssRNAv_RdRp-like (accession: cl40470) and DEAD-like helicase superfamily (accession: smart00487) conserved domains. However, the second ORF characteristic of *Fusariviridae* could not be contiguously assembled. The second transcript, 8046 nt in length, exhibited three complete ORFs, with the larger ORF containing polymerase- and helicase-related conserved domains, as expected for fusariviruses. Based on phylogenetic analysis, these sequences were designated as Dendrobium catenatum-associated fusarivirus 1 (DcFV1) and Dendrobium catenatum-associated fusarivirus 2 (DcFV2), and they were clustered with members of the genera Betafusarivirus and Alphafusarivirus, respectively (Fig. 3).

##### Lenarviricota

The *Botourmiaviridae* family comprises mono- and multi-segmented positive-sense RNA viruses that infect plants, fungi, and oomycetes [1]. Monosegmented viruses in this family have genome lengths ranging from 2 to 5 kb, while multi-segmented viruses range from 0.97 to 2.8 kb per segment. Predominantly, their replication cycle occurs in the cytoplasm, although there are reports suggesting mitochondrial replication for certain members.

**Fig. 4:**
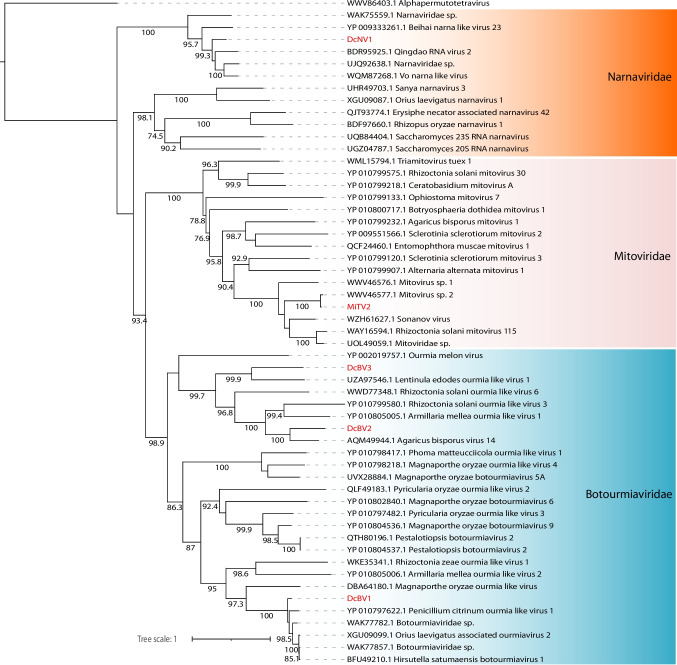
Phylogenetic reconstruction of assembled sequences related to the Lenarviricota phylum: Phylogenetic analysis, conducted using ModelTest-NG based on the Akaike information criterion (AIC), determined VT + F as the optimal evolutionary model. Bootstrap values were generated from 1000 replicates. Values below 70% are not displayed

Three assembled contigs demonstrated sequence similarity to *Botourmiaviridae* members at the amino acid level, with genome lengths of 2090 nt, 3333 nt, and 3919 nt. Complete polymerase ORFs were assembled from the two largest sequences, while the smallest contig (2090 nt) was incomplete. The two largest sequences displayed the family-unspecific ps-ssRNAv_RdRp-like conserved domain (accession: cl40470), whereas the smallest contig exhibited the ps-ssRNAv_Botourmiaviridae_RdRp domain (accession: cd23183), specific to *Botourmiaviridae*. Phylogenetic analysis revealed that these sequences clustered with unclassified botourmiaviruses, and they were subsequently named Dendrobium catenatum-associated botourmiavirus 1 to 3 (DcBV1–3) (Fig. 4).

Members of the *Narnaviridae* family are among the simplest known viruses. They are capsidless and typically consist of a non-segmented, positive-sense single-stranded RNA (ssRNA+) genome that encodes only the RNA-dependent RNA polymerase (RdRp), with genome sizes ranging from 2.3 to 3.6 kb [19]. In this study, one assembled transcript exhibited high similarity to the polymerase protein of the unclassified narnavirus Vo narna-like virus. Notably, neither the best hit nor the assembled transcript contained an RdRp conserved domain, suggesting the possibility of a truly novel viral sequence. Based on phylogenetic analysis, this transcript was designated as Dendrobium catenatum-associated narnavirus 1 (DcNV1) (Fig. 4).

##### Duplornaviricota

*Phlegiviridae* is a viral family characterized by mono-segmented, dsRNA genomes. These genomes are approximately 10.3 kbp in size and contain two primary ORFs. ORF2 encodes the RdRp, while ORF1 encodes other proteins, including a putative capsid protein and domains such as Nudix hydrolase and phytoreovirus S7. A − 1 ribosomal frameshift mechanism connects ORF1 and ORF2, potentially leading to the expression of ORF2 as part of a larger fusion polyprotein. Members of this family are known to infect fungi [39].

**Fig. 5:**
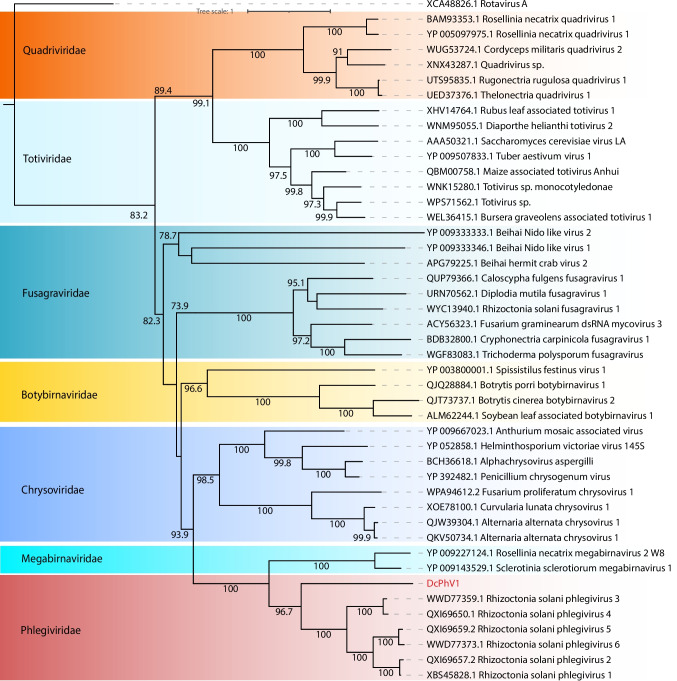
Phylogenetic reconstruction of assembled sequences related to the Duplornaviricota phylum: Phylogenetic analysis, conducted using ModelTest-NG based on the Akaike information criterion (AIC), determined Blosum62 + F as the optimal evolutionary model. Bootstrap values were generated from 1000 replicates. Values below 70% are not displayed

In the similarity search, one assembled contig displayed sequence similarity to viruses of the order Ghabrivirales, which includes families such as *Chrysoviridae*, *Megabirnaviridae*, *Quadriviridae*, *Orthototiviridae*, and *Phlegiviridae*. Specifically, its best hit Rhizoctonia solani dsRNA virus 18 (*Phlegiviridae*, genus Phlegivirus). This contig, 9045 nt in length, was assembled with an incomplete RdRp-encoding ORF and a complete capsid-related ORF. The polymerase ORF contained the conserved domain ps-ssRNAv_RdRp-like superfamily (accession: cl40470). Phylogenetic analysis revealed that this sequence is related to unclassified *Phlegiviridae* members, and it was subsequently named Dendrobium catenatum-associated phlegivirus 1 (DcPhV1) (Fig. 5).

##### Kitriniviricota

Viruses within the *Endornaviridae* family are linear, single-stranded positive-sense RNA (ssRNA+) viruses with genome sizes ranging from 9.7 to 17.6 kb. They exhibit a broad host range, infecting plants, fungi, and oomycetes. The family includes two genera: Alphaendornavirus, which infects plants, fungi, and oomycetes, and Betaendornavirus, which infects ascomycete fungi [14, 55].

**Fig. 6:**
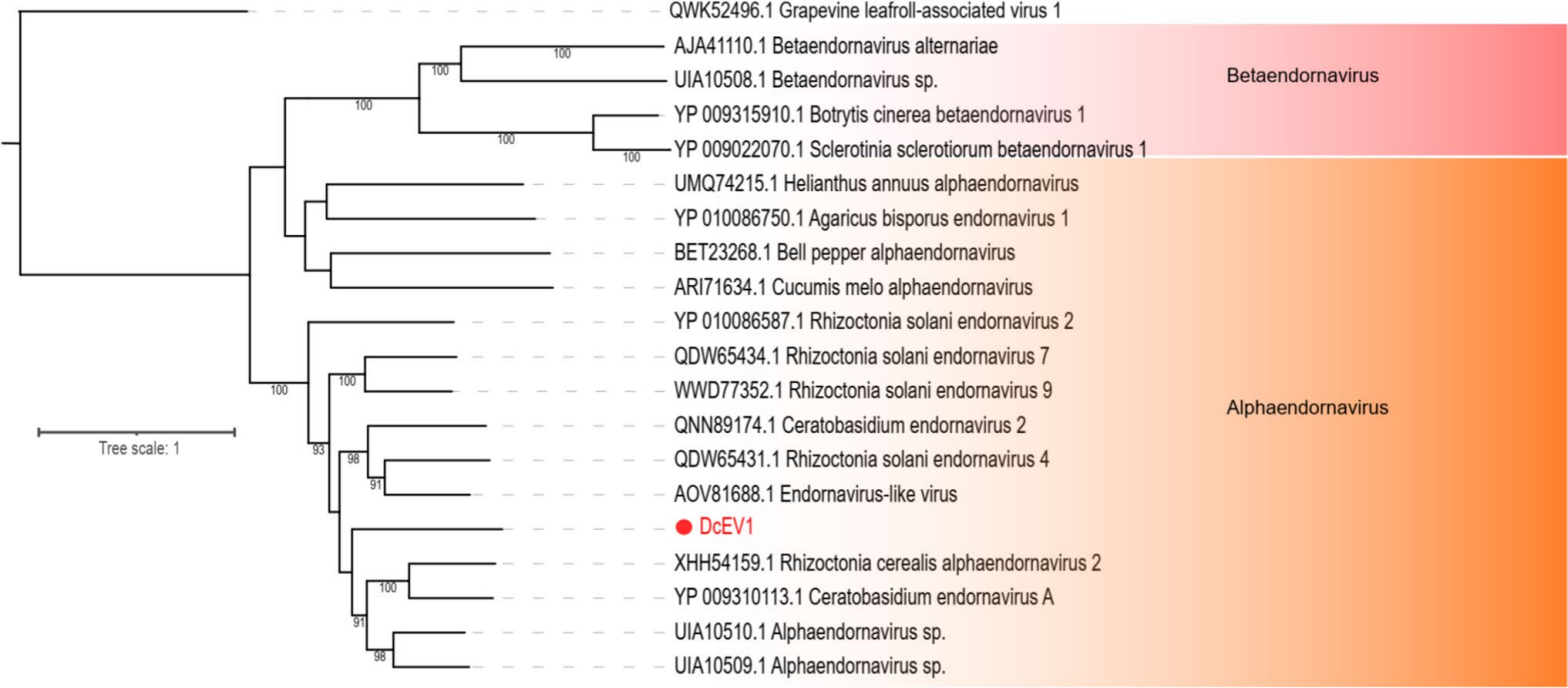
Phylogenetic reconstruction of assembled sequences related to the *Endornaviridae* family: Phylogenetic analysis, conducted using ModelTest-NG based on the Akaike information criterion (AIC), determined Blosum62 + F as the optimal evolutionary model. Bootstrap values were generated from 1000 replicates. Values below 70% are not displayed

In this study, one *Endornaviridae*-related contig was assembled, displaying amino acid sequence similarity to the polyprotein of an unclassified Alphaendornavirus. The contig, with a genome size of 4327 nt, represented an incomplete polymerase-encoding ORF that included the family-specific Endornaviridae_RdRp conserved domain (accession: cd23255). Phylogenetic analysis placed the contig within the Alphaendornavirus group. It was subsequently named Dendrobium catenatum-associated endornavirus 1 (DcEV1) (Fig. 6).

### Virus prevalence and widespread

**Fig. 7:**
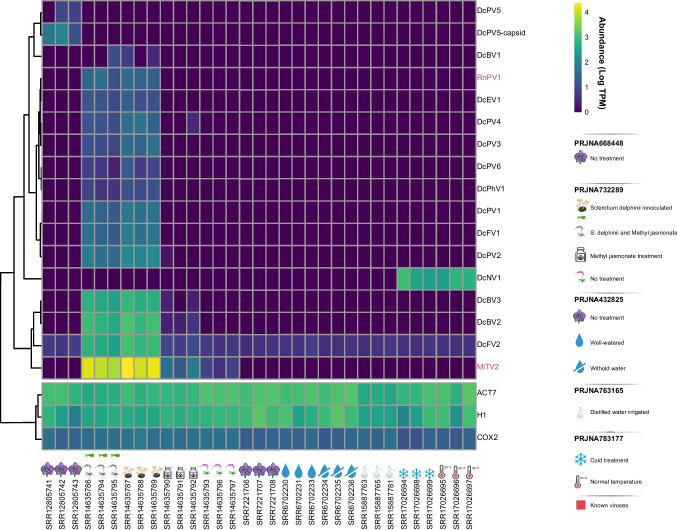
Assessment of transcriptional activity of *Dendrobium catenatum*-associated viruses. Heatmap illustrating the transcriptional activity of identified viral sequences in *D. catenatum*-derived samples. The color spectrum reflects transcription levels, ranging from low (blue) to high (yellow). Rows are clustered based on Pearson correlation. Transcriptional abundance is normalized as transcripts per million (TPM) and presented on a log10 scale. Samples are organized by project, including PRJNA668448 (*D. catenatum* without treatment), PRJNA732289 (*D. catenatum* under various treatments: *Sclerotium delphinii* inoculated, *S. delphinii* and methyl jasmonate, methyl jasmonate treatment, no treatment), PRJNA432825 (*D. catenatum* no treatment, well-watered, withhold water), PRJNA763165 (*D. catenatum* distilled water irrigated), and PRJNA783177 (*D. catenatum* cold treatment and normal temperature)

After characterizing the putative novel viral sequences, their transcriptional activity was evaluated by quantifying the viral contigs alongside constitutive host genes. Interestingly, all sequences exhibited transcriptional activity in at least six distinct libraries, with the exception of DcPV5 and DcBV1. Notably, the known virus MiTV2 and the novel DcBV2, DcBV3, and DcNV1 showed transcriptional activity levels 2 to 20 times higher than those of the constitutive host genes across all libraries where they were detected. Additionally, DcFV2 was consistently present in libraries from various projects, geographic locations, and experimental treatments, suggesting that this virus is likely a persistent member of the *Dendrobium catenatum* resident virome (Fig. 7 and Supplementary File 3).

The majority of viral sequences displayed transcriptional activity primarily in libraries from project PRJNA732289, specifically in samples infected with *Sclerotium delphinii*. This included the known virus RnPV1 and the novel viruses DcFV1, DcPhV1, DcPV1, DcPV2, DcPV3, and DcPV6. Notably, DcFV2, DcBV2, DcBV3, and MiTV2 showed a significant increase in transcriptional activity, with TPM levels in inoculated samples being, on average, 112 times higher (ranging from 200 to 16,770 TPM) than in uninfected samples (1 to 38 TPM). A similar trend was observed for DcPV4, with transcriptional activity ~11.5 times higher in inoculated samples (12 to 45 TPM) compared to non-inoculated samples (2 TPM), suggesting a strong association of these viruses with the infection state (Fig. 7 and Supplementary File 3).

Conversely, projects outside of PRJNA732289 did not yield remarkable findings. The DcPV5 virus was widespread across all three samples from project PRJNA668448, and DcNV1 displayed TPM levels approximately six times higher compared to nuclear constitutive genes in all PRJNA783177 samples. Notably, the only virus detected in both PRJNA432825, involving plants subjected to hydric stress and controls, and PRJNA763165, with plants irrigated with distilled water, was DcFV2 (Fig. 7 and Supplementary File 3).

### Codon usage bias and di-nucleotide composition in viral host adaptation

**Fig. 8:**
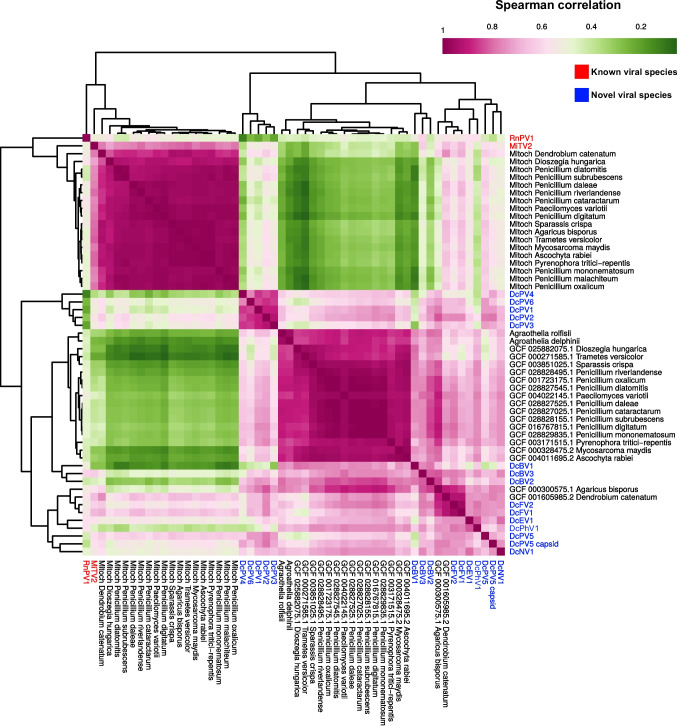
Hierarchical clustering based on Spearman’s correlation coefficients of codon and dinucleotide usage profiles in viruses and their putative host genomes. The analysis integrates both nuclear and mitochondrial genomes, aiming to uncover compositional similarities that may reflect host–virus coevolution, adaptation, or shared selective pressures. Known viral sequences are depicted in red and novel viral species are depicted in blue

A subsequent Spearman correlation analysis of dinucleotide odds ratios between viruses and microbial genomes revealed two well-defined clusters and a third, less correlated group (Fig. S2). The first cluster comprised nuclear fungal genomes alongside the viruses DcBV1 and DcBV2. Further investigation into dinucleotide and codon usage correlations indicated that members of the *Botourmiaviridae* family showed a strong compositional affinity with fungal hosts (Fig. 8). Among them, DcBV1 displayed a near-unbiased AA dinucleotide composition (Fig. 8 and Fig. S2). At the family level, only a subset of viral lineages presented sequences with relatively neutral compositions for AG, GG, GU, UG, and UU dinucleotides—patterns not observed in other families (Fig. S2).

In contrast, mitoviruses tended to associate with mitochondrial genomes, although no definitive link to plant or microbiota origins could be established (Figs. S2, S3 and S4). Partitiviruses, on the other hand, displayed diverse compositional profiles. DCPV1, 2, 3, 4, and 6 formed a coherent cluster, whereas DCPV5 diverged significantly, aligning more closely with coding sequences from *Dendrobium catenatum* and *Agaricus bisporus* (Fig. 8 and Fig. S5 and S6). This divergence was further supported by dinucleotide and codon usage patterns, as well as phylogenetic analysis (Fig. 3), suggesting that DCPV5 is more tightly associated with its host or microbiome, while other partitiviruses may infect alternative hosts or exhibit broader host ranges, as evidenced by weaker compositional correlations.

Codon usage analysis revealed a pronounced bias among partitiviruses toward AGA and, in some cases, AGG—both encoding arginine, an amino acid known to enhance protein packing and structural stability. Similar codon preferences were observed in endornaviruses and related partitiviruses. Within the clustered partitiviruses, ACG (threonine) and CGA (arginine) were also enriched. In contrast, DCPV5 exhibited a distinct codon usage pattern, favoring CCA (proline) and AGU (serine), indicating unique molecular traits (Fig. S6).

## Discussion

Metatranscriptomic profiling of *Dendrobium catenatum* revealed a diverse and dynamic microbial community, with bacteria representing the majority of sequences (67%) followed by fungi (32%), while archaea and viruses accounted for a small proportion. The bacterial assemblage was mainly composed of *Proteobacteria*, *Actinobacteria*, *Bacteroidetes*, and *Firmicutes*, with *Actinobacteria*—particularly the *Acinetobacter* genus—being especially abundant in some libraries, such as SRR17026697. The fungal fraction was dominated by *Ascomycota* and *Basidiomycota*, though its abundance and taxonomic composition varied across projects. Samples inoculated with *Sclerotium delphinii* (PRJNA732289) showed increased fungal representation, including genera such as *Aspergillus*, *Zymoseptoria*, *Botrytis*, and *Sporisorium*.

Notably, differences in microbial composition among datasets appear to reflect the influence of experimental treatments on the orchid-associated microbial community. For instance, *Firmicutes* were more abundant in control and methyl jasmonate–treated plants (PRJNA783177) but declined in samples inoculated with *S. delphinii*, suggesting that pathogen exposure may influence bacterial balance. These patterns indicate that the *D. catenatum* microbiome is both structured and responsive to biotic and environmental conditions.

Despite the fact that the RNA-seq datasets analyzed here were not originally generated with a metagenomic focus and lacked standardized microbiome-specific preparation steps, the microbial profiles observed were remarkably consistent across projects. The repeated detection of the same dominant bacterial and fungal groups, as well as the coherent shifts associated with *S. delphinii* inoculation, supports the reliability of the patterns identified. These similarities across independent datasets strengthen the confidence in our microbiome findings and indicate that the major trends reported here are robust despite methodological differences among studies.

The search for Endogenous Viral Elements (EVEs) before identifying exogenous viral elements in microbiome/virome studies is a crucial strategy to avoid false positives and ensure the accuracy of results [5]. EVEs are viral sequences that have integrated into the host genome throughout evolution, and can be mistaken for active viruses if not properly identified and excluded [12]. The identification of 7 EVEs in the genome of *Dendrobium catenatum* may provide insights into the evolutionary history of virus-host interactions in this species. Our results are in line with patterns observed in other angiosperms, such as Oryza species [7].

Exogenous viral diversity was further underscored by the identification of 16 viral species spanning seven families. Our results aligned well with previous virome studies in orchids, especially with respect to the frequent detection of *Partitiviridae* members. The co-occurrence of multiple ssRNA+ viruses, notably those in *Botourmiaviridae* and *Fusariviridae*, supported the notion that unique evolutionary pressures operate in *D. catenatum*, potentially driven by its specialized ecological niche and complex symbiotic interactions with fungal endophytes [30, 51, 57, 58].

The virome associated with *Dendrobium catenatum* comprised a diverse array of mycoviruses, including DcEV1 (*Endornaviridae*), DcBV1–3 (*Botourmiaviridae*), DcNV1 (*Narnaviridae*), DcFV1–2 (*Fusariviridae*), and DcPV1–6 (*Partitiviridae*). This spectrum of viral taxa underscored the complexity of plant–fungus–virus interactions in orchids. Notably, endornaviruses (e.g., DcEV1) are found in both plant and fungal systems and have been associated with conferring stress tolerance or growth advantages, further hinting at their capacity to influence host physiology in a beneficial manner [9]. Botourmiaviruses (DcBV1–3) and narnaviruses (DcNV1) identified in our study mirrored similar viruses detected in phytopathogenic fungi, where reductions in virulence or alterations in host metabolism have been noted [10, 66], suggesting that these viruses might also contribute to attenuating fungal pathogenicity in orchid-associated communities. Among the most intriguing are the partitiviruses (DcPV1–6), a group well-documented for inducing hypovirulence in fungal pathogens such as *Rosellinia necatrix* and *Sclerotinia sclerotiorum*. Their ability to decrease fungal growth and disrupt virulence factors has made them promising candidates for biocontrol applications [49, 65].

Mycoviruses have demonstrated the capacity of alter host phenotypes, leading to reduced fungal growth and hypovirulence. Interestingly, we detected a contig related to Rosellinia necatrix partitivirus 1-W8, a virus whose presence in fungal isolates is correlated with decreased growth, abnormal colony morphology, and hypovirulence [63]. Comparable effects are documented in other systems, such as Sclerotinia sclerotiorum partitivirus 1 (SsPV1/WF-1), raising the possibility that similar mechanisms may be at play in the orchid-associated mycobiome. Of note, the exact fungal hosts of the viruses remain to be confirmed through molecular or culture-based approaches.

The increase in viral transcriptional activity detected in samples inoculated with *Sclerotium delphinii* (project PRJNA732289) appears to be of particular interest. Several viruses, including MiTV2, DcBV2, DcBV3, and DcFV2, showed higher TPM values in inoculated plants compared to control conditions, suggesting that pathogen exposure may influence the expression or abundance of these viral sequences. This observation may reflect (i) changes in the metabolic or transcriptional activity of fungal endophytes responding to the pathogen challenge, (ii) fluctuations in the abundance of potential mycoviral hosts due to interactions with *S. delphinii*, or (iii) stress-induced modulation of viral expression within the orchid–fungus system.

Additionally, some partitiviruses (DcPV1, DcPV2, DcPV3, DcPV5) and RnPV1 were detected primarily in inoculated samples, which could indicate a closer association with the pathogenic fungus or with fungal partners that respond to pathogen-induced stress. The hypovirulence-related effects of partitiviruses documented in other fungal species [49, 63] suggest that similar interactions could occur within the *D. catenatum* microbiome, although further evidence is needed. Future work combining fungal isolation, co-cultivation experiments, and virus detection assays will be essential to clarify the ecological and functional roles of these potential mycoviruses in orchid-associated microbial communities.

Our compositional analyses, based on dinucleotide odds ratios and codon usage patterns, provided additional insight into potential virus–host associations. Hierarchical clustering based on Spearman’s correlation coefficients revealed a distinct group comprising nuclear fungal genomes together with DcBV1 and DcBV2, suggesting that these *Botourmiaviridae* members may infect fungi and have coevolved under host-specific selective pressures [27]. In contrast, mitoviruses consistently grouped with mitochondrial genomes, consistent with their known persistence in these organelles [65]. Partitiviruses exhibited greater diversity: while DCPV1, 2, 3, 4, and 6 clustered closely together, DCPV5 diverged, showing compositional affinities with coding sequences from *Dendrobium catenatum* and *Agaricus bisporus*. This divergence, supported by both codon and dinucleotide patterns as well as phylogenetic analyses, indicates that DCPV5 may maintain a stronger association with *D. catenatum*, whereas other partitiviruses could have broader host ranges. At the codon level, a bias toward AGA and AGG—both coding for arginine—was observed among most partitiviruses, an amino acid associated with enhanced protein packing and stability, possibly reflecting adaptation to host translational environments [17]. In contrast, DCPV5 exhibited enrichment for CCA (proline) and AGU (serine), suggesting distinct molecular characteristics that may underlie specialized interactions with its host [24, 47].

Beyond compositional trends, these findings illustrate the complex interplay between plants, fungi, and their associated viruses. Increasing evidence supports the occurrence of horizontal virus transfer (HVT) between plant viruses and mycoviruses, implying that such exchanges can shape host fitness and ecosystem dynamics [33, 45, 46]. In *D. catenatum*, these relationships are particularly intriguing given the orchid’s dependence on fungal symbionts for germination and nutrient uptake [50, 68]. Moreover, mycoviruses that induce hypovirulence in phytopathogenic fungi have shown potential as biocontrol agents, offering sustainable alternatives to chemical management [15, 37]. Other studies also indicate that mycoviruses may enhance fungal competitiveness through mechanisms such as toxin production or modulation of host metabolism [32, 64]. Together, these observations suggest that mycovirus-mediated interactions could play a role in shaping the orchid holobiont, while also providing a promising avenue for future biotechnological applications.

## Conclusion

In this study, we employed a metatranscriptomic approach to explore the microbial and viral communities associated with *Dendrobium catenatum*, revealing a complex interplay between the plant, its fungal associates, and a diverse array of RNA viruses. Through analysis of public RNA-seq datasets, we identified members of several viral families, many of which are typically linked to fungal hosts. Codon usage and dinucleotide composition analyses further suggested patterns of adaptation between viruses and their putative hosts, while some viral sequences clustered closely with known mycoviruses, suggesting possible co-evolutionary dynamics or horizontal transmission. Our findings expand current knowledge of orchid-associated viromes and point to *D. catenatum* as a rich and understudied reservoir of viral diversity, underscoring the importance of integrative omics approaches in understanding the ecological and evolutionary roles of viruses within plant holobionts.

However, this study is based solely on in silico analyses of public RNA-seq data, which limits the ability to definitively assign viral sequences to specific hosts or confirm active infections. Future research should focus on experimental validation—such as isolating fungal endophytes, confirming viral presence through RT-PCR or in situ hybridization, and conducting co-culture or transmission assays—to verify host specificity and infection dynamics. Additionally, controlled metatranscriptomic surveys across different developmental stages and environmental conditions of *D. catenatum* could provide deeper insights into the factors shaping its virome. Such integrative molecular and ecological approaches will be crucial to fully elucidate the role of mycoviruses in orchid–fungus–virus interactions and their potential applications in biocontrol and plant health.

## Supplementary Information

ESM 1 Supplementary Materials: The following supporting information can be downloaded at the journal website. XXX, Fig. S1: Abundance of virus-derived transcripts under different conditions; Fig. S2: Dinucleotide frequency profiles of viral, mitochondrial, and nuclear genomes derived from reference fungal species and *Dendrobium catenatum* transcriptomic data; Fig. S3: Hierarchical clustering based on Spearman’s correlation coefficients of dinucleotide usage profiles in viruses and their putative host genomes; Fig. S4: Hierarchical clustering based on Spearman’s correlation coefficients of codon usage profiles in viruses and their putative host genomes (DOCX 712 kb)

ESM 2 Supplementary File 1: Libraries information. (XLSX 14 kb)

ESM 3 Supplementary File 2: BLASTn and BLASTx similarity searchs results. (XLSX 55 kb)

ESM 4 Supplementary File 3: Transcriptional activity of putative viral sequences. (CSV 7936 kb)

ESM 5 Supplementary File 4: Codon usage bias and di/tri nucleotide usage reference sequences. (XLSX 8 kb)

ESM 6 Supplementary File 5: Metagenomic analyses results. (XLSX 1542 kb)

ESM 7 Supplementary File 6: Characterization of Endogenous viral elements. (XLSX 45 kb)

## Data Availability

The assembled sequences are currently deposited at NCBI nucleotide database under accession codes BK75138-BK75154 and are also available at the Supplementary File 2
