# Supplementary material for: Metaviromic insights into the viral community associated with Dendrobium catenatum
Source: Braz J Microbiol. 2026 Jan 28;57(1):43. doi: 10.1007/s42770-025-01816-5 (PMC12852558; doi:10.1007/s42770-025-01816-5)
Supplement: Supplementary file 1 — Supplementary Materials: The following supporting information can be downloaded at the journal website. XXX, Fig. S1: Abundance of virus-derived transcripts under different conditions; Fig. S2: Dinucleotide frequency profiles of viral, mitochondrial, and nuclear genomes derived from reference fungal species and Dendrobium catenatum transcriptomic data; Fig. S3: Hierarchical clustering based on Spearman’s correlation coefficients of dinucleotide usage profiles in viruses and their putative host genomes; Fig. S4: Hierarchical clustering based on Spearman’s correlation coefficients of codon usage profiles in viruses and their putative host genomes (DOCX 712 kb) [file 42770_2025_1816_MOESM1_ESM.docx]

**Supporting information for:**

**Metaviromic Insights into the Viral Community Associated with *Dendrobium* *catenatum***

**Rogério Mercês Ferreira Santos ^1,†,*^, Lucas Yago Melo Ferreira ^2,†^, João Pedro Nunes Santos², Lucas Barbosa de Amorim Conceição^2^ , Giovanna Venas Oliveira^1^, Cassio van den Berg^1^ and Eric Roberto Guimarães Rocha Aguiar^3,*^**

|  |
| --- |

^1^ Data Processing and Bioinformatics Laboratory (BIOINFO), Department of Biological Sciences (DCBIO), Universidade Estadual de Feira de Santana (UEFS), Avenida Transnordestina, No Number, Novo Horizonte, Feira de Santana, Bahia, Brazil, 44036-900

^2^ Center of Biotechnology and Genetics, Department of Biological Sciences, Universidade Estadual de Santa Cruz (UESC), Rodovia Jorge Amado, km 16, Ilhéus, Bahia, Brazil, 45662-900

^3^ Postgraduate program in Computational Modeling in Science and Technology, Department of Engineering and Computing, Universidade Estadual de Santa Cruz (UESC), Ilhéus-BA, Brazil

^†^ These authors contributed equally to this work.

***** Correspondence: e-mail@e-mail.com; Tel.: (optional; include country code; if there are multiple corresponding authors, add author initials)

**
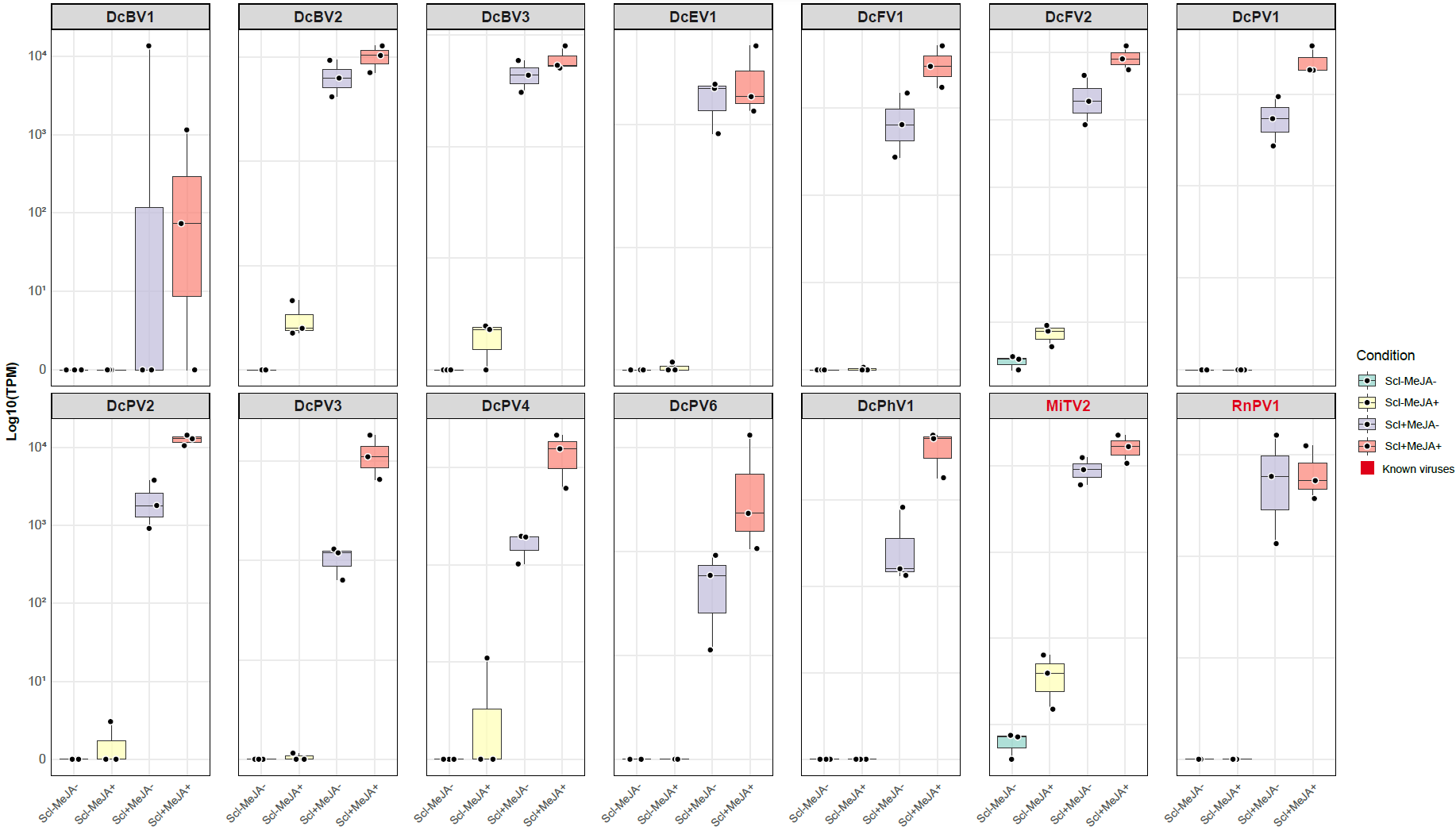
** **Figure S1: Abundance of virus-derived transcripts under different conditions.** Boxplots display the TPM abundance of transcripts representing *Dendrobium catenatum*-associated viruses across four treatments: (1) inoculated with *Sclerotium delphinii* (Scl+MeJA-), (2) inoculated with *S. delphinii* and treated with methyl jasmonate (MeJA) (Scl+MeJA+), (3) treated only with MeJA (Scl-MeJA+), and (4) control without inoculation or MeJA treatment (Scl-MeJA-). The Y-axis indicates normalized TPM abundance, while the X-axis categorizes the different treatment groups. Statistical analysis was conducted using the Wilcoxon test, with significance determined at a p-value < 0.05.


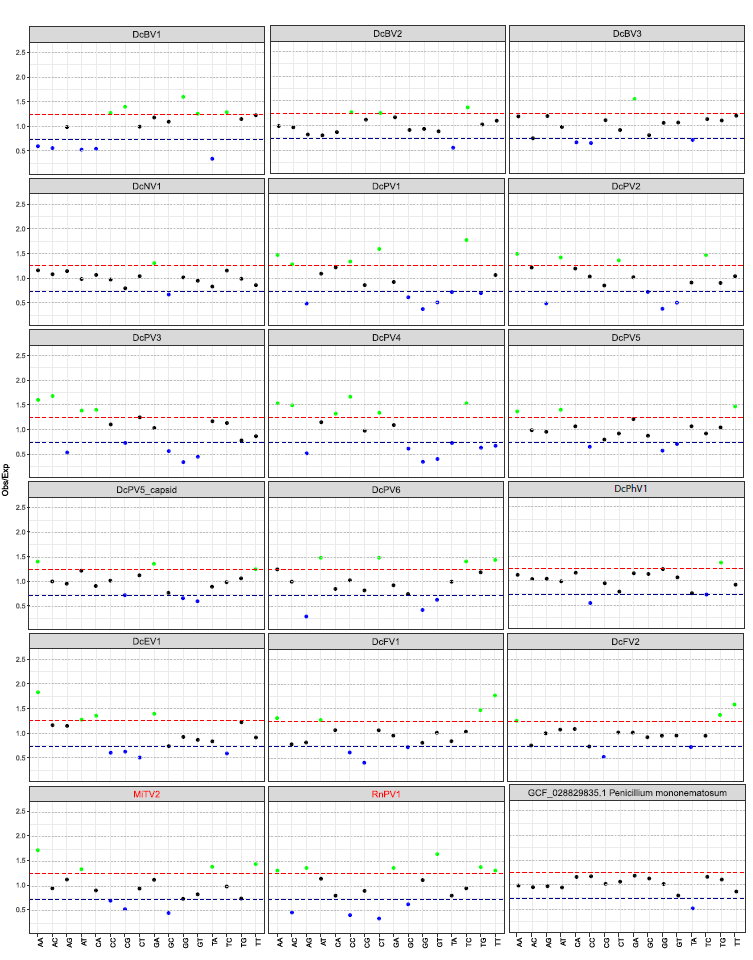


**Figure S2**: **Dinucleotide frequency profiles of viral and nuclear genome derived from reference fungal species.** Green circles denote dinucleotide odds ratios that are significantly biased relative to expected frequencies, while blue circles indicate ratios within the unbiased range. Dashed lines represent the defined thresholds for unbiased values, set between 0.78 and 1.25. Known viral species are depicted in red.


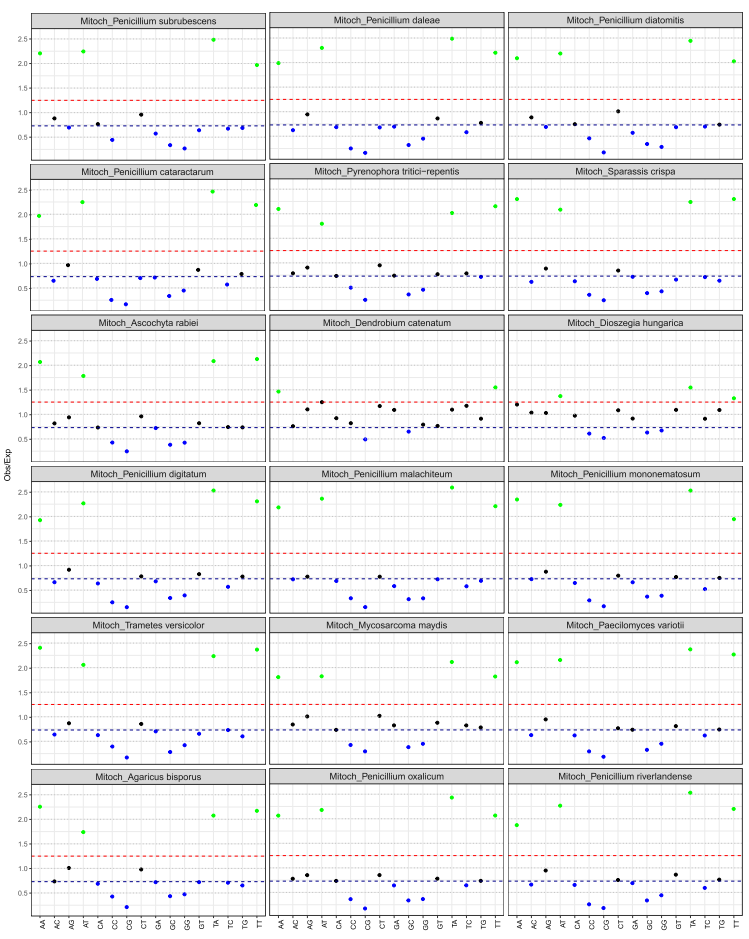


**Figure S3**: **Dinucleotide frequency profiles of mitochondrial genomes from reference fungal species.** Green circles denote dinucleotide odds ratios that are significantly biased relative to expected frequencies, while blue circles indicate ratios within the unbiased range. Dashed lines represent the defined thresholds for unbiased values, set between 0.78 and 1.25.


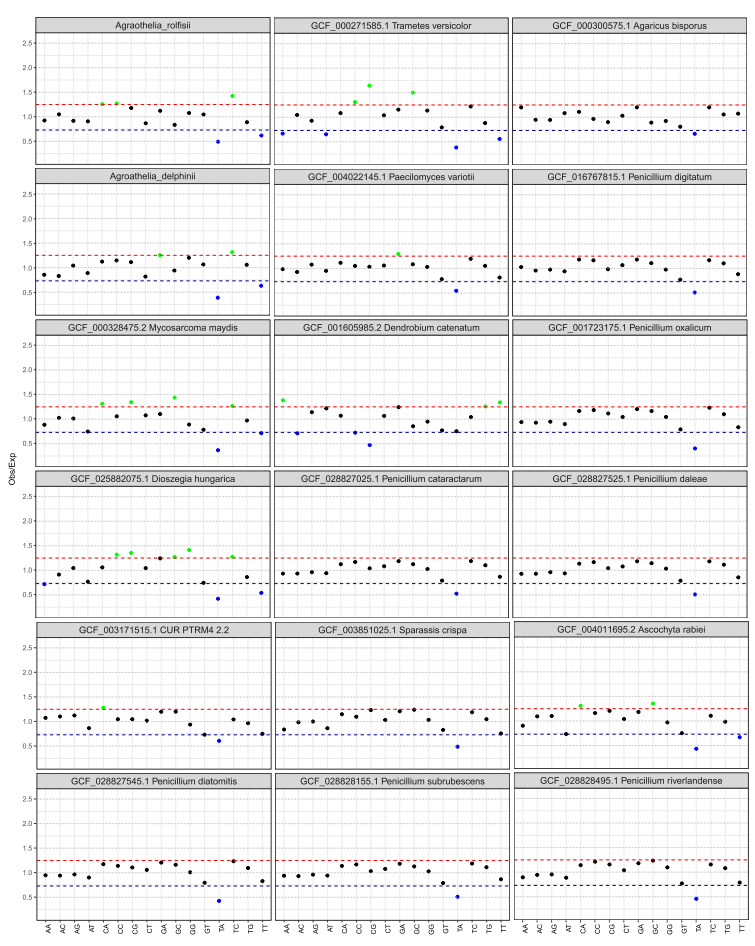


**Figure S4**: **Dinucleotide frequency profiles of nuclear genomes derived from reference fungal species.** Green circles denote dinucleotide odds ratios that are significantly biased relative to expected frequencies, while blue circles indicate ratios within the unbiased range. Dashed lines represent the defined thresholds for unbiased values, set between 0.78 and 1.25.


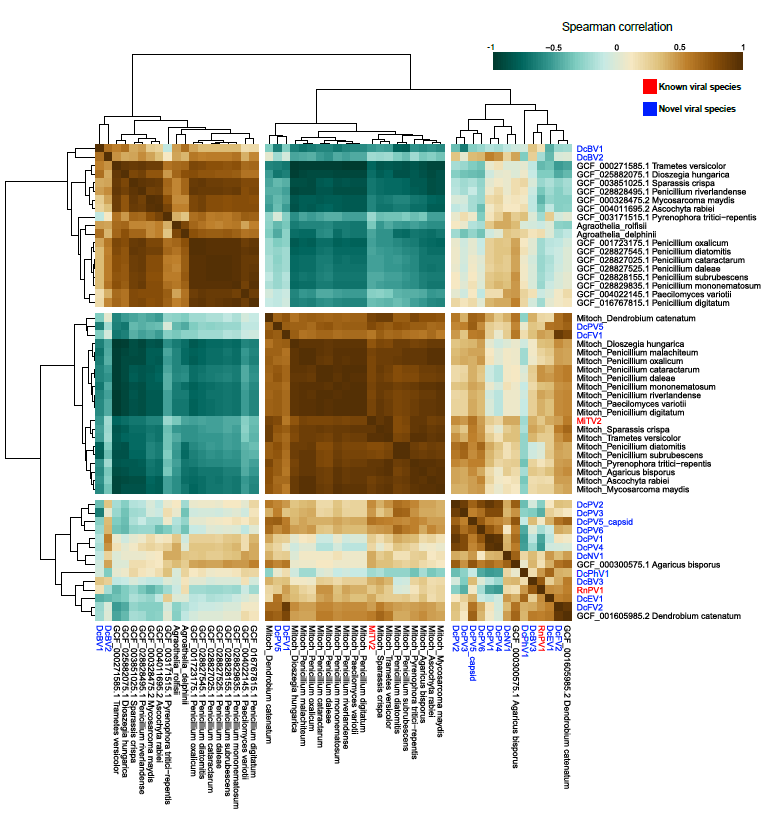


**Figure S5**: **Hierarchical clustering based on Spearman's correlation coefficients of dinucleotide usage profiles in viruses and their putative host genomes.** The analysis includes both nuclear and mitochondrial genomic components, highlighting compositional similarities that may reflect evolutionary or functional associations between viruses and their potential hosts. Known viral sequences are depicted in red and novel viral species are depicted in blue.


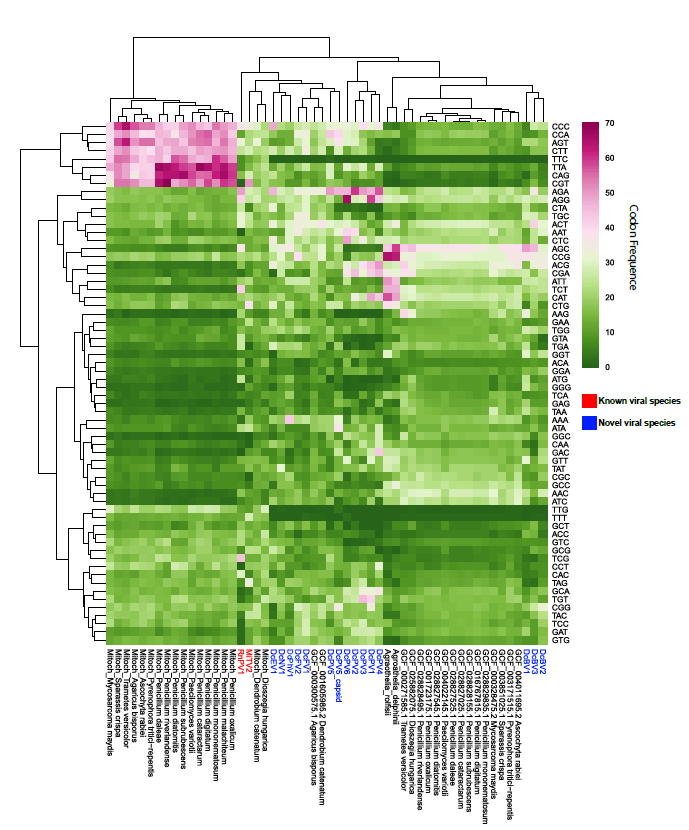


**Figure S6**: **Hierarchical clustering based on Spearman’s correlation coefficients of codon usage profiles in viruses and their putative host genomes.** The analysis integrates both nuclear and mitochondrial genomes, aiming to uncover compositional similarities that may reflect host–virus coevolution, adaptation, or shared selective pressures. Known viral sequences are depicted in red and novel viral species are depicted in blue.
